# Production of an enzymatic cocktail by *Aspergillus japonicus* in a stirred-tank bioreactor for application in animal feed

**DOI:** 10.1007/s11274-026-04839-8

**Published:** 2026-04-17

**Authors:** Ana Lorena de Oliveira Simas, Aline Pereira Almeida, Nathalia Nunes Glienke, Quézia de Melo Santana, Rodrigo Mattos Silva Galeano, Charles Kiefer, Karina Márcia Ribeiro de Souza Nascimento, Maria de Lourdes Teixeira de Moraes Polizeli, Douglas Chodi Masui, Fabiana Fonseca Zanoelo, Giovana Cristina Giannesi

**Affiliations:** 1 Programa Multicêntrico de Pós-Graduação em Bioquímica e Biologia Molecular, Sociedade Brasileira de Bioquímica e Biologia Molecular (SBBq), Universidade Federal de Mato Grosso do Sul, Campo Grande, MS Brazil; 2 Laboratório de Bioquímica Geral e de Microrganismos (LBq), Instituto de Biociências (INBIO), Universidade Federal de Mato Grosso do Sul, Campo Grande, MS 79070-900 Brazil; 3 Departamento de Biologia, Faculdade de Filosofia, Ciências e Letras de Ribeirão Preto, Universidade de São Paulo, Ribeirão Preto, SP 14040-901 Brazil; 4 Faculdade de Medicina Veterinária e Zootecnia (FAMEZ), Universidade Federal de Mato Grosso do Sul, Campo Grande, MS Brazil

**Keywords:** Animal diet, Bench-scale bioreactor, Enzymes, Submerged cultivation, Synergism

## Abstract

New enzymatic sources need to be explored to meet the increasing demand for bioactives used in the supplementation of non-ruminant animals. Phytase and xylanase are widely used in animal nutrition due to their ability to degrade phytic acid and xylan, respectively. This study aimed to (i) produce phytase and xylanase by culturing *Aspergillus japonicus* on medium containing wheat bran + soybean bran (1:1 w/w) as alternative carbon sources in a stirred-tank bioreactor and (ii) compare this process with that conducted in Erlenmeyer flasks. The resulting enzymatic cocktails and commercial enzymes were evaluated for their ability to hydrolyze poultry diets (D1–D9). The production peaks of phytase and xylanase in the bioreactor occurred at 120 h (842.30 ± 43.62 U/L) and 144 h (3211.29 ± 274.98 U/L), respectively. After 144 h of cultivation, protein concentration in the bioreactor was approximately twice that observed in Erlenmeyer flasks. ECPAjBr displayed the highest hydrolytic activity for inorganic phosphate in diet D6 (42.34 ± 2.20 mg/g), followed by diet D7 (38.67 ± 0.46 mg/g). Regarding reducing sugars, ECXAjBr exhibited the highest activity in diets D1 and D8, releasing 147.71 ± 18.18 mg/g and 145.83 ± 11.39 mg/g, respectively. All tested diets, regardless of their composition, indicated a synergistic interaction between commercial enzymes and those present in the *A. japonicus* enzyme cocktail, which exhibits a broad spectrum of hydrolytic activities, including high levels of accessory enzymes. The application of this cocktail in animal diets holds great potential for increasing nutrient bioavailability in non-ruminant animal production.

**Graphical abstract:**

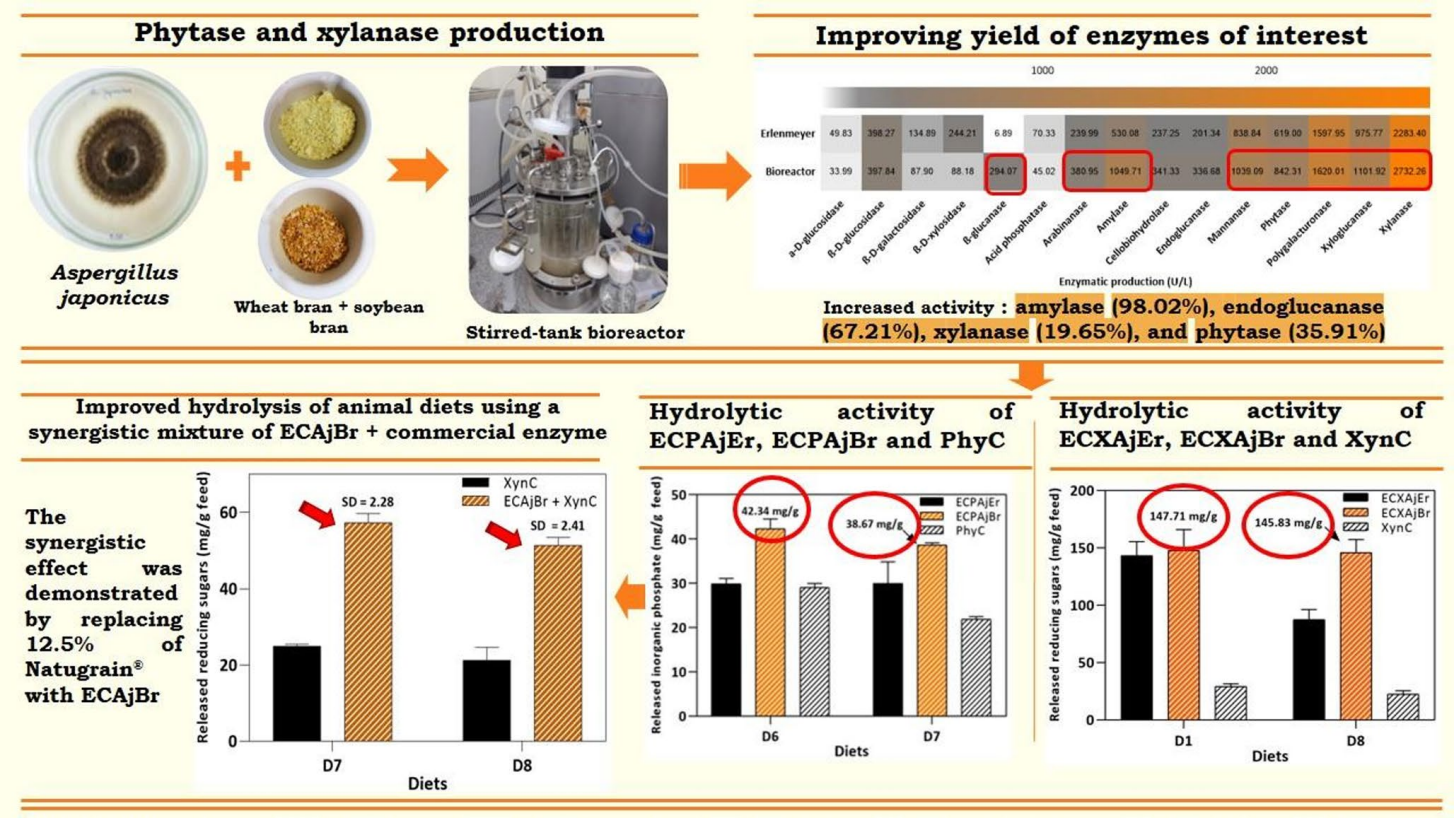

## Highlights

Phytase and xylanase production by *Aspergillus japonicus* in a stirred-tank bioreactor.

Fungal cells were cultured on a mixture of wheat bran + soybean bran as carbon sources.

The fungal enzyme cocktail showed higher hydrolytic capacity than commercial enzymes.

The cocktail showed synergism with commercial enzymes in hydrolyzing animal diets.

## Introduction

The global animal feed enzyme market was valued at US$1.32 billion in 2023 and is projected to grow at a compound annual growth rate of 5.0% from 2024 to 2030. This growth is driven by the rapid expansion of the livestock sector, which has increased the demand for enhanced nutrition and improved diet digestibility. Furthermore, population growth, the scarcity of high-quality ingredients, and stricter government regulations regarding the quality of animal production have encouraged farmers to incorporate enzyme supplements into animal diets to compensate for nutritional deficiencies, ensure animal health, and achieve optimal performance (Grand View Research 2024).

A nutritious and easily digestible diet is necessary to achieve high animal yields. Animal diets are mainly composed of corn and soybean, although sunflower meal, barley, wheat, sorghum, and oat can also be used as energy-rich sources (Plouhinec et al. 2023). However, the presence of anti-nutritional factors, such as phytic acid, non-starch polysaccharides, and tannins, limits the use of these ingredients. Exogenous enzymes, including phytase and xylanase, have shown beneficial effects in animal feeding by reducing the levels of anti-nutritional factors (Sureshkumar et al. 2023; Perera et al. 2025). Additionally, these enzymes increase the bioavailability of mineral nutrients, thereby enhancing intestinal health and improving animal growth (Sureshkumar et al. 2023; Tomé-Rodríguez et al. 2025).

Phytases, or myo-inositol hexakisphosphate phosphohydrolases, belong to the group of phosphatases responsible for catalyzing the degradation of phytic acid, promoting the release of phosphate ions (PO_4_^2−^) (Sureshkumar et al. 2023; Singh et al. 2024). The gastrointestinal system of non-ruminant animals has limited capacity to degrade phytate due to the absence of endogenous phytases and the low abundance of microorganisms capable of digesting this phosphorus compound. Consequently, the poor absorption of phosphorus leads to its excretion and the need for supplementation with inorganic phosphorus. Another strategy is to supplement animal diets with phytases, which reduces phosphorus excretion and minimizes the environmental impacts of this pollutant (Sureshkumar et al. 2023). Of note, several studies demonstrated that the addition of phytase to animal diets promotes phosphorus release, both in vitro and in vivo (de Oliveira Simas et al. 2025; Priya et al. 2025; Tomé-Rodríguez et al. 2025).

Xylanases, or endo-β-1,4-xylanases, are part of the glycosyl hydrolase group, encompassing different families listed on the CAZy platform (www.cazy.org). These enzymes hydrolyze β-d-xylosidic bonds in the xylan backbone, facilitating the breakdown of non-starch polysaccharides, which have anti-nutritional effects due to their resistance to digestive enzymes (Plouhinec et al. 2023). Thus, xylanases reduce the viscosity of animal feed along the digestive tract of animals and increase nutrient accessibility, consequently improving gut health and growth performance. It should also be noted that hemicellulases such as xylanases exhibit synergistic activity with cellulases in the degradation of the plant cell wall, representing interesting additives in animal nutrition (Hu et al. 2011; Singh et al. 2015; Sruthi et al. 2023). In addition to xylanase and phytase, which are widely used in animal feed, carbohydrases, such as cellulase, β-mannanase, β-glucanase, xylanase, and galactosidase, as well as other classes of enzymes, including proteases, lipases, and phosphatases, also play an important role in diets for non-ruminant animals, contributing to increased protein digestibility and feed energy (Sureshkumar et al. 2023).

An essential step in industrial enzyme production is the scaling up of fungal cultivation. Submerged fermentation has been widely used for this purpose due to the ease of controlling process parameters (Fernandes et al. 2024). This type of fermentation can be carried out in stirred-tank bioreactors, both on a laboratory and on an industrial scale. This reactor type prevents the formation of dead zones during mixing, which create concentration gradients and limit oxygen transfer (Cachon et al. 2019). Overall, stirred-tank bioreactors are easy to operate, achieve good homogenization between enzyme and substrate, enable uniform transfer, and provide efficient control of reaction conditions (Xia et al. 2021; Wang et al. 2025).

*Aspergillus japonicus* has great potential for the production of hydrolytic enzymes and enzyme cocktails with application in various industrial sectors, as demonstrated in previous research conducted by our group (da Silva et al. 2019; de Alencar Guimarães et al. 2024; de Oliveira Simas et al. 2025). However, few studies have investigated enzyme production by *A. japonicus* in bioreactors, representing a significant gap in the literature, particularly regarding the concomitant production of phytase and xylanase. In light of the foregoing, this study aimed to produce phytase and xylanase by culturing *A. japonicus* in a stirred-tank bioreactor using agro-industrial wastes as alternative carbon sources. Another objective was to assess the hydrolytic capacity of the resulting enzyme cocktail on poultry diets and its potential synergism with commercial enzymes.

## Materials and methods

### Fungal strain and growth conditions

*A. japonicus* Saito (UFMS 48.136) was isolated from soil at the Private Reserve of Natural Heritage of the Federal University of Mato Grosso do Sul (UFMS), Campo Grande, Mato Grosso do Sul, Brazil. Native to the Brazilian Cerrado, this fungal strain was identified and registered in GenBank under the accession code MW587714 by da Silva et al. (2019). Fungal cells were cultured on potato dextrose agar medium (HiMedia, Mumbai, India) at 30 °C for 5–8 days and preserved on silica gel beads.

### Effect of reactor type and fermentation time on enzyme production

Submerged cultivation was carried out in 250 mL Erlenmeyer flasks containing 40 mL of Mandels’ (1976) medium. The medium composition was as follows (w/v): urea (0.30), (NH_4_)_2_SO_4_ (1.40), KH_2_PO_4_ (0.50), MgSO_4_·7H_2_O (0.30), CaCl_2_·2H_2_O (0.30), and peptone (0.75). Additionally, it contained 0.5% of the following salt solutions per liter: ZnSO_4_·7H_2_O (280 µL), MnSO_4_·H_2_O (320 µL), FeSO_4_·7H_2_O (1 mL), and CoCl_2_ (400 µL). The pH was adjusted to 6.0. Flasks were inoculated with 10 mL of a fungal suspension containing 10^6^ spores/mL. The medium was supplemented with 1% wheat bran/soybean bran (1:1 w/w) as carbon sources, according to de Oliveira Simas et al. 2025. Cultures were incubated for 24 to 144 h at 30 °C. Then, aliquots were collected and centrifuged at 4 °C and 10.000 × *g* for 10 min. The supernatant was used as crude enzyme extract.

A pelletized cell pre-inoculum was prepared for submerged cultivation in the stirred-tank bioreactor (Liu and Wilkins 2020). Briefly, 5 mL of a spore suspension containing 10^6^ spores/mL was inoculated in a 125 mL Erlenmeyer flask containing 30 mL of modified Mandels’ medium (pH 6.0) supplemented with 1% wheat bran/soybean bran (1:1 w/w), equivalent to 1% (v/v) of the bioreactor working volume; pH was standardized according to de Oliveira Simas et al. (2025). The culture was incubated in a shaker at 110 rpm and 30 °C for 24 h. Subsequently, the inoculum was directly poured into the bioreactor.

Three different experimental conditions were evaluated, in which parameters such as pH, dissolved oxygen, aeration, and agitation were varied. The stirred-tank bioreactor used in the experiments was a BioFlo^®^/CelliGen^®^ 310 reactor (Eppendorf) with a working volume of 3 L. The bioreactor vessel was fitted with Rushton-type radial impellers. The temperature was maintained at 30 °C. Mettler-Toledo pH and dissolved-oxygen probes were used to monitor pH and dissolved oxygen, respectively. Batch fermentations were performed using modified Mandels’ medium (pH 6.0) containing 1% (w/v) wheat bran + soybean bran (1:1 w/w) as carbon sources. Antifoam Prodooze^®^ AE (Prodooze Biotech) was added at 100 µL/L at the beginning of cultivation, which is within the range commonly recommended for microbial fermentations. This concentration was selected to efficiently suppress foam formation caused by aeration and protein secretion, while minimizing any potential interference with microbial growth, enzyme expression, or downstream processing.

The medium pH was adjusted to 6.0 by the addition of 0.4 M H_2_SO_4_ or 0.5 M NaOH. Subsequently, the fermentations were started by inoculating the microorganism. The stirring speed was set at 100–500 rpm. Airflow was provided by continuous injection (0.75–4 vvm) of compressed air passed through a sterile filter. Fermentation runs were performed as outlined in Table 1. Samples were collected between 24 and 144 h of fermentation to assess enzyme production. Then, the aliquots were centrifuged at 4 °C and 10.000 × *g* for 10 min. The supernatant was used as crude enzyme extract containing the extracellular enzymes.

**Table 1:** Process parameters evaluated during phytase and xylanase production by *Aspergillus japonicus* in a stirred-tank bioreactor

Parameter	Run 1	Run 2	Run 3
Initial pH	6.0 (setpoint 5.5–6.5)	6.0 (monitored only)	6.0 (monitored only)
Dissolved oxygen	50% with gas cascade	Monitored only	50% with gas cascade
Agitation	100–500 rpm	100 rpm	100–500 rpm
Aeration	0.75–4 vvm	1 vvm	1 vvm
Fermentation duration	96 h	96 h	144 h

### Enzymatic assays

First, extracellular enzyme activities were measured using natural substrates (in parentheses): amylase (starch, Megazyme^®^), β-glucanase (β-glucan, Megazyme^®^), arabinanase (debranched arabinan, Megazyme^®^), xylanase (beechwood xylan, Sigma–Aldrich^®^), endoglucanase (carboxymethylcellulose, Sigma–Aldrich^®^), mannanase (locust bean, Sigma–Aldrich^®^), polygalacturonase (polygalacturonic acid sodium salt, Sigma–Aldrich^®^), and xyloglucanase (xyloglucan, Megazyme^®^). The activities were determined by quantifying reducing sugars by the 3,5-dinitrosalicylic acid (DNS) method (Miller 1959). The assay mixture consisted of 30 µL of a 1% (w/v) substrate solution in distilled water, 40 µL of 100 mM sodium acetate buffer (pH 5.0), and 30 µL of enzyme extract. The exception was xylanase, for which 100 mM citrate–phosphate buffer (pH 5.0) was used (McIlvaine, 1921). The reaction was stopped by adding 100 µL of DNS. Absorbance was measured at 540 nm on a SpectraMax 384 Plus spectrophotometer (Molecular Devices, USA). Reducing sugars were quantified using standard curves for arabinose, glucose, mannose, galacturonic acid, and xylose (1 mg/mL). One unit of enzyme activity was defined as the amount of enzyme required to release 1 µmol of reducing sugars per minute. Activities are expressed in U/L.

Enzyme activities were also determined against the following synthetic substrates (in parentheses): α-d-glucosidase (*p*-nitrophenyl-α-d-glucopyranoside, Sigma–Aldrich^®^), β-d-galactosidase (*p*-nitrophenyl-β-d-galactopyranoside, Sigma–Aldrich^®^), β-d-glucosidase (*p*-nitrophenyl-β-d-glucopyranoside, Sigma–Aldrich^®^), β-d-xylosidase (*p*-nitrophenyl-β-d-xylopyranoside, Sigma–Aldrich^®^), acid phosphatase (*p*-nitrophenyl-β-d-phosphate, Sigma–Aldrich^®^), and cellobiohydrolase (*p*-nitrophenyl-β-d-cellobioside, Sigma–Aldrich^®^). The assay mixture consisted of 30 µL of a 10 mM substrate solution in distilled water, 30 µL of 100 mM sodium acetate buffer (pH 5.0), and 40 µL of enzyme extract. After 15 min, 100 µL of 0.5 M Na_2_CO_3_ was added to stop the reaction. Absorbance was measured at 410 nm on a SpectraMax 384 Plus spectrophotometer (Molecular Devices, USA) using a standard curve of *p*-nitrophenol (1 mM). One unit of enzyme activity was defined as the amount of enzyme required to release 1 µmol of *p*-nitrophenol per minute per milliliter, with activities expressed in U/L.

Phytase activity was determined according to the method of Heinonen and Lahti (1981). For the reaction, 250 µL of 100 mM sodium acetate buffer (pH 5.0) was incubated with 10 mM phytic acid sodium salt hydrate (Sigma–Aldrich^®^) and 50 µL of enzyme extract for 10 min at 50 °C. The reaction was stopped by adding 1.5 mL of stop solution (10 mM ammonium molybdate, 5 M sulfuric acid, and acetone at a 1:1:2 ratio) and 100 µL of 1 M citric acid. Absorbance was measured at 355 nm using a SpectraMax 384 Plus spectrophotometer (Molecular Devices, USA). A 1 mM KH_2_PO_4_ solution was used as standard. One unit of enzyme activity was defined as the amount of enzyme required to release 1 µmol of product per minute, with activities expressed in U/L.

Protein quantification was performed using the Bradford method (1976), using 200 µg/mL bovine serum albumin as standard. Absorbance was measured at 595 nm using a spectrophotometer. Results are expressed in mg protein/L.

### Hydrolytic capacity of *A. japonicus* enzyme cocktails and commercial enzymes on animal diets

Hydrolytic assays were conducted using 0.2 g of poultry diet. The following poultry diets were tested: D1, rice grits + soybean bran; D2, oat + soybean bran + corn; D3, soybean hulls + soybean bran + corn; D4, dried distillers grains with solubles (DDGS) + corn; D5, rice bran + soybean bran + corn; D6, wheat bran + soybean bran + corn; D7, millet + millet rice + soybean bran; D8, corn + soybean meal; and D9, sorghum + soybean bran. The quantity and composition of the diets were standardized and described according to de Oliveira Simas et al. (2025). Of note, fungal enzyme extracts and commercial preparations of phytase (PhyC, Natuphos©) and xylanase (XynC, Natugrain©) were standardized in enzymatic units before use in hydrolytic experiments.

For analysis of hydrolytic capacity, 0.2 g of diet sample was placed in 2 mL Eppendorf tubes. Xylanase and its commercial counterpart (XynC) were added at 100 U, whereas phytase and its commercial counterpart (PhyC) were added at 10 U. The enzymatic cocktail produced in Erlenmeyer flasks and used to evaluate inorganic phosphate release was designated ECPAjEr, whereas that produced in the bioreactor was named ECPAjBr. Similarly, for reducing sugars released from the diets, the enzymatic cocktail produced in Erlenmeyer flasks was labeled ECXAjEr, and that produced in the bioreactor was defined as ECXAjBr. The reaction systems were adjusted to a final volume of 2 mL with 100 mM sodium acetate buffer (pH 5.0). Enzymatic hydrolyses were conducted at 40 °C under shaking at 150 rpm for 24 h.

After the incubation period, samples were centrifuged at 14.000 × *g* for 2 min. The supernatants were collected for analysis of released products. Reducing sugars released from the diets was measured using 10 µL of sample, 90 µL of distilled water, and 100 µL of DNS reagent, as described by Miller (1959). The inorganic phosphorus released from the diets was determined using 10 µL of sample, 300 µL of stop solution, and 20 µL of citric acid, according to the method proposed by Heinonen and Lahti (1981). The effect of the enzyme cocktail phytase and xylanase-rich and accessory enzymes was analyzed by calculating the change in the release of reducing sugars and inorganic phosphate. Results are expressed in mg/g feed.

### Evaluation of the synergism between *A. japonicus* enzymes and a commercial enzyme

Additional hydrolytic reactions were conducted to investigate the potential synergistic activity between the *A. japonicus* enzyme cocktail produced under bioreactor conditions and a commercial xylanase (Natugrain©). The control consisted of 200 U of commercial xylanase. An experimental treatment was prepared by replacing 12.5% of the commercial enzyme, resulting in a mixture containing 175 U of XynC and 25 U of xylanase from the *A. japonicus* enzymatic cocktail. Thus, both samples contained the same enzyme load (200 U). Treatments were compared based on the total amount of reducing sugars released. Reactions were conducted as described in the previous section, using animal diets. The occurrence of synergism between enzymes was evaluated by calculating the degree of synergism (Eq. 1), according to the equation proposed by Hu et al. (2011):

1 $$DS=\frac{{\sum}_{}^{}XynC+ECAjBr}{{\sum}_{}^{}XynC}$$

where DS is the degree of synergism, ECAjBr is the total amount of reducing sugars released by the enzyme cocktail produced by *A. japonicus* under bioreactor conditions, and XynC is the total amount of reducing sugars released by the commercial enzyme. According to the definition, enzymatic interactions presenting DS index values greater than 1.0 are classified as synergistic (Hu et al. 2011).

### Statistical analysis

All experiments were performed in triplicate (*n* = 3), and results are presented as mean ± standard deviation. Means were compared between treatments using one-way analysis of variance (ANOVA), followed by Tukey’s test. Differences were considered significant at *p* < 0.05. Statistical analyses were performed using GraphPad Prism version 8.

## Results

### Enzyme production under bioreactor conditions

This study presents the first investigations into the production of a phytase- and xylanase-rich extracellular enzyme complex by *A. japonicus* in a stirred-tank bioreactor using wheat bran and soybean bran (1:1) as carbon sources. Considering the lack of reports in the literature on the production of these enzymes by *A. japonicus* in bioreactors, the production process was standardized using three operational models. These operational modes were designated runs 1, 2, and 3, and were tested to identify the optimal cultivation conditions for enzyme production (Table 1).

In bioreactor systems, several variables influence mass transfer and mixing. Some of the most important variables are agitator speed and gas flow rate (Salmon et al. 2016). Therefore, these parameters were varied among runs. During the bioreactor cultivation, a cascade control strategy was employed to maintain DO at 50% saturation (runs 1 and 3). In this control scheme, for example, agitation speed was used as the primary control variable, while the aeration rate was adjusted if necessary. Under these conditions, the target DO level of 50% was achieved and maintained by increasing agitation up to 300 rpm, and no further increase in aeration rate was required. The results demonstrated a strong influence on enzyme production.

Some reaction conditions were adopted from the study of Liu and Wilkins (2020), who cultured *Aspergillus nidulans* using a 1% (v/v) cell pellet inoculum. The fungal morphology remained predominantly in the form of compact pellets throughout the cultivation. In runs 1 and 2, this pelletized morphology was maintained for the entire fermentation period. In contrast, in run 3, signs of sporulation began to be observed after 96 h of cultivation. Temperature and initial pH followed the parameters used in Erlenmeyer flask cultures.

**Fig. 1:**
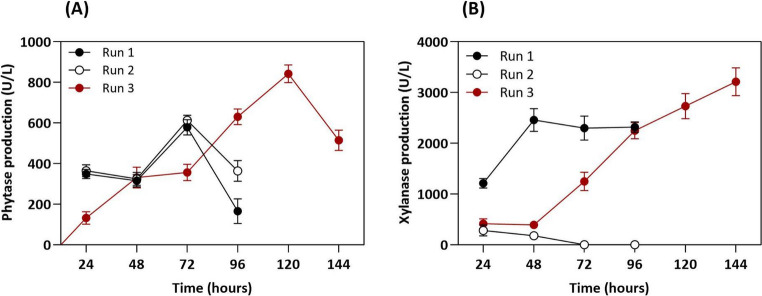
Time-dependent production of (**A**) phytase and (**B**) xylanase by *Aspergillus japonicus* in a stirred-tank bioreactor. The culture medium was supplemented with 1% wheat bran + soybean bran (1:1 w/w) as alternative carbon sources

In run 1 of the bioreactor assay, the pH was controlled (setpoint 5.5–6.5) and the fermentation was aerated. *A. japonicus* exhibited peak phytase activity (587.68 ± 37.45 U/L) at 72 h. However, after the peak, phytase production dropped sharply. Run 2 was designed to improve enzyme production yield. In this run, the pH was only monitored, without active control. Additionally, agitation was reduced to 100 rpm, a condition previously recommended by Maller et al. (2014) for phytase production. In this run, peak phytase production was also observed at 72 h, reaching 613.14 ± 24.93 U/L. Subsequently, with the adjustments made in run 3, the phytase production peak occurred at 120 h, reaching 842.30 ± 43.62 U/L. This result represents a 27.20% increase compared with previous conditions (Fig. 1A).

For xylanase production, in run 1, the enzyme peak (2459.68 ± 226.26 U/L) was reached after 48 h. However, in run 2, xylanase production was drastically reduced, becoming almost undetectable. Michelin et al. (2013) observed that the optimal agitation speed for xylanase production ranges from 300 to 400 rpm, given the high levels of dissolved oxygen. A low oxygen concentration in the broth acts as a limiting nutrient, indicating the need for higher oxygen input to support growth and enzyme production. Therefore, agitation and aeration significantly influence xylanase production by fungal cultures (Michelin et al. 2013; Iram et al. 2022). On the basis of these recommendations, the dissolved oxygen concentration was maintained at 50% in run 3, with the agitation adjusted between 100 and 500 rpm as needed; whereas, it was observed that a maximum agitation speed of 300 rpm was sufficient to effectively control the DO. With these adjustments, the xylanase production peak occurred at 144 h, reaching 3211.29 ± 274.98 U/L (Fig. 1B).

### Evaluation of enzyme production by *A. japonicus* in Erlenmeyer flasks and a bioreactor

**Fig. 2:**
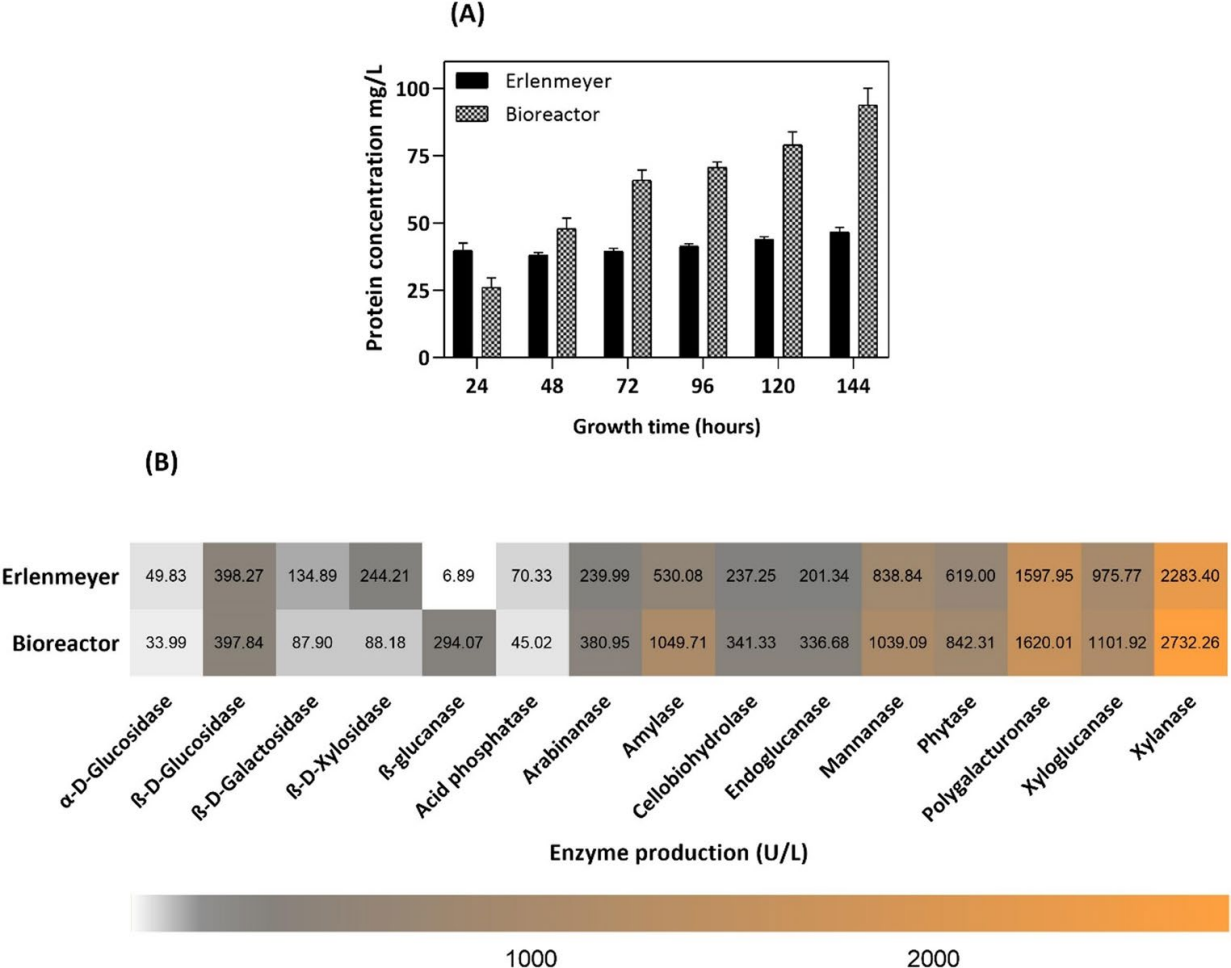
Comparison of (**A**) protein and (**B**) enzyme production by *Aspergillus japonicus* in Erlenmeyer flasks and a stirred-tank bioreactor. Fungal cells were cultured in Mandels’ medium (1976) supplemented with 1% wheat bran + soybean bran (1:1 w/w) as alternative carbon sources

Protein production was analyzed for comparison between small-scale cultivation in 250 mL Erlenmeyer flasks and a bench-top bioreactor during the initial stages of optimization. After 144 h of cultivation, the protein concentration in the bioreactor (93.80 ± 6.25 mg/L) was approximately twice that observed in Erlenmeyer flasks (46.63 ± 1.68 mg/L), as shown in Fig. 2A.

The composition of the enzyme cocktails produced in both cultivation systems was analyzed. The analysis focused on extracellular enzymes related to the degradation of lignocellulosic biomass, such as cellulases, hemicellulases, amylolytic enzymes, and phosphorus-releasing enzymes. In total, 15 enzymes were quantified, including phytase and xylanase. Results are expressed in U/L (Fig. 2B). Several enzymes showed higher production under bioreactor conditions than under flask conditions, particularly amylase (1049.71 ± 177.80 U/L, 98.02% increase), endoglucanase (336.67 ± 72.22 U/L, 67.21% increase), arabinanase (380.95 ± 338 U/L, 58.73% increase), cellobiohydrolase (341.32 ± 11.34 U/L, 43.86% increase), phytase (842.30 ± 43.62 U/L, 35.91% increase), and xylanase (2732.25 ± 247.74 U/L, 19.65% increase). 

### In vitro hydrolytic capacity of *A. japonicus* enzyme cocktails produced under Erlenmeyer flask and bioreactor conditions on animal diets

The action of *A. japonicus* enzymes on animal diets was investigated by assessing the release of inorganic phosphate and reducing sugars, considered indicative of nutrient degradation capacity. The same enzyme concentrations were used for assays with cocktails produced in Erlenmeyer flasks and the bioreactor. The enzymatic cocktails contained 10 U of phytase, designated ECPAjEr (Erlenmeyer flask) and ECPAjBr (bioreactor), and 100 U of xylanase, designated ECXAjEr (Erlenmeyer flask) and ECXAjBr (bioreactor).

**Fig. 3:**
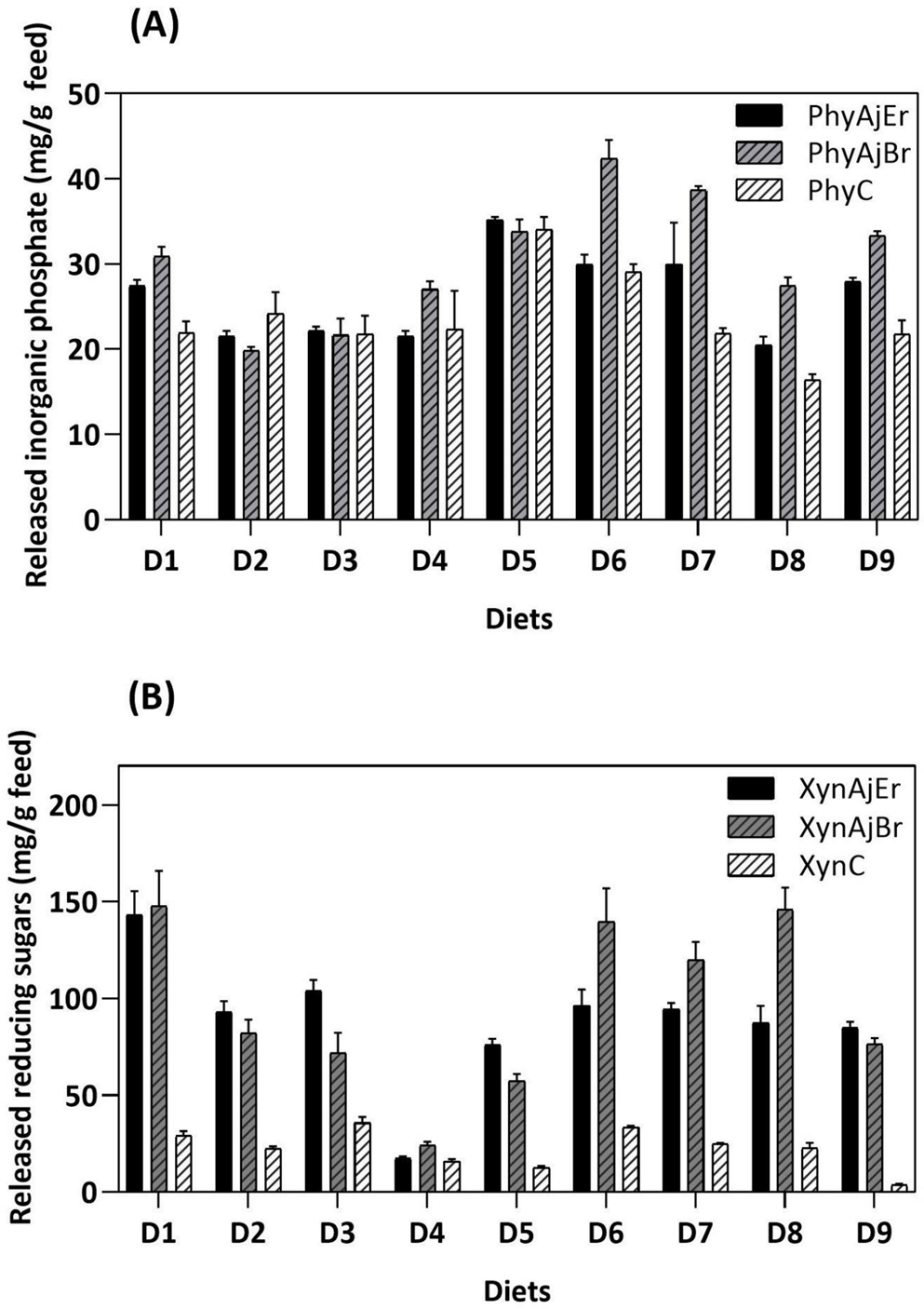
(**A**) Inorganic phosphate and (**B**) reducing sugars released from animal diets by an enzyme cocktail produced by *Aspergillus japonicus* in Erlenmeyer flasks and in a stirred-tank bioreactor, compared with commercial enzymes. (**A**) and (**B**) The abbreviations denote phytase and xylanase from *A. japonicus* produced in Erlenmeyer flasks (PhyAjEr and XylAjEr, respectively) and in a bioreactor (PhyAjBr and XynAjBr, respectively) and in commercial enzymes (PhyC and XynC, respectively). Assays were performed at pH 5.0 and 40 °C for 24 h (*n* = 3). D1, rice grits + soybean bran diet; D2, oat + soybean bran + corn diet; D3, soybean hull + soybean bran + corn diet; D4, dried distillers grains with solubles + corn diet; D5, rice bran + soybean bran + corn diet; D6, wheat bran + soybean bran + corn diet; D7, millet + millet rice + soybean bran diet; D8, corn + soybean meal diet; D9, sorghum + soybean meal diet

Overall, a slight improvement in diet degradation was observed, likely due to the higher protein concentration in the bioreactor-produced cocktail. Comparison between ECPAjEr and ECPAjBr indicated that the latter showed superior performance, although ECPAjEr and the commercial enzyme (PhyC) achieved similar results in some diets. Regarding inorganic phosphate release, ECPAjBr showed the highest hydrolytic activity in D6 (42.34 ± 2.20 mg/g), corresponding to a 29.33% higher activity than that of ECPAjEr, followed by D7 (38.67 ± 0.46 mg/g), representing a 22.52% increase relative to ECPAjEr (Fig. 3A).

Xylanases derived from the enzymatic cocktails (ECXAjEr and ECXAjBr) and the commercial xylanase (XynC) were evaluated for their ability to release reducing sugars. It should be noted that the commercial xylanase also exhibits other enzyme activities, such as amylase (71.58 U), endoglucanase (3.55 U), β-glucanase (4.64 U), and β-glucosidase (3.00 U) activities. No pectinase activity was detected. Notably, ECXAjBr was highly effective in hydrolyzing D1 and D8, releasing 147.71 ± 18.18 mg/g and 145.83 ± 11.39 mg/g reducing sugars, respectively. XynC, on the other hand, exhibited the best performance in diets D3 (35.69 mg/g) and D6 (33.45 ± 0.80 mg/g). Also noteworthy, ECXAjBr outperformed ECXAjEr in D8, providing a 40% higher release of reducing sugars, followed by D6, with a 30.97% higher release (Fig. 3B).

### Evaluation of the synergistic activity of *A. japonicus* enzymatic cocktail and Natugrain^®^ in the in vitro hydrolysis of animal diets

The synergistic effect between the commercial enzyme Natugrain^®^ and the enzymatic cocktail from *A. japonicus* obtained by bench-scale bioreactor production (ECAjBr) was evaluated by replacing 12.5% of the commercial enzyme with ECAjBr. Even with this low substitution ratio, all tested diets showed a degree of synergy greater than 1 (Table 2). The greatest changes in sugar release were observed for D8 (from 21.25 to 51.38 mg/g) and D7 (from 25.02 to 57.26 mg/g). These findings suggest a synergistic interaction between the commercial enzyme and the fungal enzyme cocktail, as reported by Hu et al. (2011).

**Table 2:** Synergism between the commercial enzyme mixture Natugrain^®^ and an enzyme cocktail from *Aspergillus japonicus* (ECAjBr) in releasing reducing sugars from animal diets

Diet	Released reducing sugars (mg/g)		Degree of synergism
	Natugrain^®^	Natugrain^®^ + ECAjBr	
D1	37.77 ± 2.13	39.36 ± 0.10^ns^	1.04
D2	22.50 ± 1.01	42.65 ± 4.11****	1.89
D3	35.69 ± 3.08	44.44 ± 2.82****	1.24
D4	12.54 ± 0.76	17.61 ± 0.42**	1.40
D5	17.26 ± 1.93	30.95 ± 3.19****	1.79
D6	31.90 ± 2.04	53.73 ± 0.95****	1.68
D7	25.02 ± 0.42	57.26 ± 2.45****	2.28
D8	21.25 ± 3.41	51.38 ± 2.06****	2.41
D9	5.55 ± 1.13	8.43 ± 2.95^ns^	1.51

Assays were performed at pH 5.0 and 40 °C for 24 h (*n* = 3). Asterisks indicate significant differences from the control by Tukey’s test. ** *p* < 0.01; **** *p* < 0.0001; ns, not significant. D1, rice grits + soybean bran diet; D2, oat + soybean bran + corn diet; D3, soybean hull + soybean bran + corn diet; D4, dried distillers grains with solubles + corn diet; D5, rice bran + soybean bran + corn diet; D6, wheat bran + soybean bran + corn diet; D7, millet + millet rice + soybean bran diet; D8, corn + soybean meal diet; D9, sorghum + soybean meal diet

## Discussion

Alternative carbon sources are considered biotechnological tools for obtaining valuable products, such as enzymes, from lignocellulosic materials, thereby reducing costs in industrial settings (Singh et al. 2021). Different nutrient-rich carbon sources have been used to optimize cultivation conditions and maximize enzyme production in various cultures, such as *Aspergillus niger* on corn cobs (Michelin et al. 2013)d *niger* on DDGS (Iram et al. 2022), as well as *Ganoderma* sp. MR-56 on wheat bran (Salmon et al. 2016), and *Trichoderma longibrachiatum* LMBC 172 and *Thermothelomyces thermophilus* LMBC 162 on tamarind seeds (Contato et al. 2021).

Due to their rich nutrient composition, wheat bran and soybean bran were used as alternative carbon sources in enzyme production assays. These ingredients were reported to be effective inducers of phytase and xylanase production (de Oliveira Simas et al. 2025). Wheat bran is composed, on average, of 55% to 60% non-starch carbohydrates, 14% to 25% starch, 13% to 18% protein, 3% to 8% minerals, and 3% to 4% lipids (Hell et al. 2014). Soybean bran contains approximately 40% protein, 20% lipids, 35% carbohydrates, and 5% minerals (Ha et al. 2009). This nutritional composition likely contributes to their efficiency as enzyme inducers.

Previous studies with *A. japonicus* have reported high levels of xylanase and phytase production in in vitro assays, which is associated with the efficient degradation of animal feed components (de Oliveira Simas et al. 2025). However, no study had previously described the simultaneous production of phytase and xylanase in a bioreactor system. In the present study, a significant enhancement in the production of xylanase and phytase, as well as of other hydrolytic enzymes, was observed when the fungus was cultivated on a medium containing wheat bran + soybean bran (1:1) in a stirred-tank bioreactor. Furthermore, the hydrolytic activity of the resulting enzyme extract on poultry feed ingredients was markedly higher than that observed with Erlenmeyer flask cultures or commercial enzymes.

The operational parameters applied differently among the three runs directly influenced phytase and xylanase production. In run 1, the cultivation was carried out with active control of dissolved oxygen (50%) using a gas cascade, with agitation varying between 100 and 500 rpm and adjustable aeration (0.75–4 vvm), in addition to pH control within the range of 5.5–6.5. However, strict pH control may act as a limiting factor, as it can interfere with the fungal metabolic dynamics throughout the cultivation (Germec and Turhan 2023). In run 2, the process parameters were maintained under more static conditions, without dissolved oxygen control, with fixed agitation at 100 rpm, constant aeration at 1 vvm, and pH only monitored. These conditions resulted in lower oxygen transfer and reduced operational flexibility, which may have limited fungal metabolism and, consequently, enzyme production (Antecka et al. 2016; Machado et al. 2022) leading to inferior results compared to those observed in the subsequent run.

The best enzyme production was observed in run 3, when the pH was monitored but not controlled and the agitation (100–500 rpm) was allowed to vary freely as needed to maintain the levels of dissolved oxygen, with a 50% gas cascade and 1 vvm aeration. Run 3 showed superior performance in phytase production, with increases of 30.22% and 27.19% compared to runs 1 and 2, respectively. Regarding xylanase production, an increase of 23.40% was observed compared to run 1. These results suggest that this strategy enabled the maintenance of adequate oxygenation conditions throughout the cultivation, favoring both fungal growth and enzyme secretion. In addition, the maintenance of a predominantly pelletized morphology up to approximately 96 h, followed by the onset of sporulation, may be associated with metabolic adjustments occurring during the late stages of cultivation, (Veiter et al. 2018) which could contribute to changes in enzyme secretion profiles.

Increasing aeration generally enhances the levels of dissolved oxygen during the growth phase, favoring microbial development and enzyme synthesis. In the present study, higher oxygen levels (50%) in the bioreactor were associated with increased phytase and xylanase activities, as well as greater protein accumulation (101.5% higher than when cultivated in Erlenmeyer). Dissolved oxygen is a key parameter, as variations in oxygen availability can profoundly affect cellular physiology and metabolism (Michelin et al. 2013; Abdella et al. 2020). As observed in other studies with similar microorganisms, such as *A. niger* C-6, enzyme production is highly influenced by dissolved oxygen and, consequently, by aeration and agitation (García-Kirchner et al. 2005).

Peak phytase production by *A. japonicus* under optimized conditions occurred after 120 h of cultivation (842.30 ± 43.62 U/L), in agreement with previous results reported in the literature. Coban et al. (2015) studied phytase production by *Aspergillus ficuum* in a bioreactor with 1 L of working volume under controlled pH (4.5), 0.9 vvm aeration, and 300 rpm agitation. A maximum activity of 3.45 U/mL was observed after 120 h of cultivation. By contrast, cultivation of *Ganoderma* sp. at an initial pH of 6.0 without further pH control resulted in a maximum phytase activity of 14.5 U/mL after 72 h.

The maximum xylanase production observed in the present study occurred at similar or earlier times (144 h of cultivation, 3211.29 ± 274.98 U/L) than those reported in the literature. For instance, *A. niger* NRRL 330 showed optimal fermentation performance between 168 and 216 h of cultivation at 310 rpm and 1.4 vvm, which increased cellulase activity from 0.6 to 0.82 U/mL and xylanase activity from 3.99 to 52.76 U/mL compared with non-optimized conditions (Iram et al. 2022). In another study, *A. nidulans* achieved a maximum xylanase production of 1250 U/mL at 2 vvm and 400 rpm after 96 h of cultivation in batch mode (Abdella et al. 2020).

Germec and Turhan (2023) investigated whether pH control influences enzyme production. The absence of active pH control showed a positive effect on enzyme synthesis, a finding that was also observed in the present study. Similarly, Salmon et al. (2016) reported that pH control can negatively affect phytase production, suggesting that the addition of HCl and/or NaOH to maintain a constant pH may lead to a decline in enzyme yield over time. Based on the results obtained in the current study, it can be inferred that strict pH regulation is not necessary and may even hinder enzyme productivity (El Enshasy et al. 2018; Germec and Turhan 2023). Overall, the optimization of cultivation conditions, particularly regarding aeration rate, agitation, and pH control or its absence, has proven to be an effective strategy to enhance enzyme activity levels, as demonstrated by several studies (García-Kirchner et al. 2005; Michelin et al. 2013; Salmon et al. 2016; Liu and Wilkins 2020; Abdella et al. 2020; Iram et al. 2022; Germec and Turhan 2023).

The primary advantage of using bioreactors lies in the possibility of scaling up the production of the enzymes of interest, thereby enabling their future industrial application. It is well established that changes in scale and culture medium volume can influence both the profile and yield of enzyme production (Pinheiro et al. 2020; Contato et al. 2021). In this context, *A. japonicus* cultivation in a bioreactor yielded twice the protein concentration obtained in Erlenmeyer flasks, indicating an increase in enzyme production under stirred-tank bioreactors conditions. The enhancement can be attributed to factors such as improved control and availability of aeration, dissolved oxygen, and other operational parameters. The detected proteins predominantly correspond to extracellular enzymes involved in substrate degradation for subsequent fungal assimilation. This interpretation is supported by the absence of extensive mycelial disruption, as well as the lack of extraction or quantification of intracellular proteins, reinforcing that the analyzed proteins are mainly enzymatic and secreted in nature.

Contato et al. (2021) studied enzyme production by comparing total protein production in Erlenmeyer flasks and stirred-tank bioreactors using tamarind seeds as carbon source. Bioreactor conditions provided a 70.5% increase (from 18.20 ± 1.57 to 25.80 ± 1.91 µg/mL) in protein production by *Trichoderma longibrachiatum* LMBC 172 and a 74.5% increase (from 21.30 ± 1.64 to 28.60 ± 2.03 µg/mL) by *Thermothelomyces thermophilus* LMBC 162 compared with Erlenmeyer flask cultivation. Using a similar approach, Pinheiro et al. (2020) reported that *Trametes versicolor* produced significantly more laccase and total proteins in a stirred-tank bioreactor than in Erlenmeyer flasks, with laccase activity increasing by about 143%. These results demonstrate the advantages of scaling up to improve protein concentration, with important implications for future studies and further process development.

The composition of the enzyme cocktail produced by microorganisms depends directly on the culture medium and cultivation conditions, as well as on the carbon source used as substrate (García-Kirchner et al. 2005; Plouhinec et al. 2023). For lignocellulosic biomass degradation, for example, enzymes such as amylases, cellulases, hemicellulases, pectinases, among others, are widely employed in animal nutrition (Plouhinec et al. 2023). In addition, enzymes related to the phosphorus cycle, such as other phosphatases, have also been investigated with a focus on zootechnical applications (Valente Junior et al. 2024).

Given that the enzymes of interest of this study, namely phytase and xylanase, are widely used in animal nutrition, a cocktail enriched with these enzymes and with accessory enzymes could be particularly advantageous for applications in animal feed. The production of other enzymes in a bioreactor was also evaluated, observing an increase in protein production compared to cultivation in Erlenmeyer flasks, especially of amylase, endoglucanase, arabinase, and cellobiohydrolase. Amylase is responsible for the hydrolysis of starch into smaller sugars, contributing to the digestion of polysaccharides present in plant-based ingredients (Sureshkumar et al. 2023). Endoglucanase and cellobiohydrolase, in turn, are cellulases that act synergistically as part of an enzymatic complex involved in cellulose degradation (Plouhinec et al. 2023). Arabinanases, on the other hand, are hemicellulolytic enzymes that catalyze the hydrolysis of arabinan, a polysaccharide rich in arabinose and a component of hemicellulose and pectins, playing an important role in digestion and nutrient assimilation in the animal digestive tract (Seiboth and Metz 2011).

The *A. japonicus* enzymatic cocktail produced at flask and bench-reactor scales demonstrated a superior capacity to hydrolyze poultry diets compared with commercial enzymes. Moreover, it showed superior performance than literature reports, particularly in terms of phosphorus and sugar release, which are important nutrients for animal nutrition. In a previous study, phytase from *Penicillium oxalicum* demonstrated the ability to improve the nutritional value of feed through the time-dependent release of inorganic phosphate (Priya et al. 2025). The highest release of inorganic phosphate (3.986 mg/g) was obtained at 37 °C using 200 U of phytase in 0.5 g of feed for 48 h.

Numerous studies examined the dephosphorylation of wheat flour, as this is a common ingredient in animal and human nutrition. Phytase from *Aspergillus oryzae* SBS50 achieved high releases of inorganic phosphorus from wheat flour (10.42 mg/g) after 24 h of reaction, followed by millet flour (9.10 mg/g), chickpea flour (6.2 mg/g), and rice flour (3.9 mg/g) (Pragya 2023). *Aspergillus fumigatus* phytase released 9.1 mg/g inorganic phosphate from 10 g of wheat flour after 30 min of reaction (Thakur et al. 2022). Similarly, phytase from *Humicola nigrescens* was incubated with 10 g of wheat flour for 72 h and released 0.65 mg/g phosphate (Bala et al. 2014). A phytase cocktail produced by *Rhizopus oligosporus* released 0.82 mg/g inorganic phosphate after being incubated with 5.0 g of wheat flour for 24 h (Suresh and Radha 2015).

Wu et al. (2022) reported high sugar release from different substrates using varying concentrations of xylanase EX1 from *A. niger* (Bestzyme Bio-products^®^): 86.28 mg/g from DDGS using 54 U/g xylanase, 65 mg/g from corn straw using 45 U/g xylanase, and 190 mg/g from wheat bran using 15 U/g xylanase. Interestingly, Dahiya et al. (2021) observed synergy between xylanase (100 U/g) and phytase (15 U/g) from *Myceliophthora thermophila*. The enzymes were applied to poultry feed, releasing high levels of reducing sugars (58.58 mg/g feed) and inorganic phosphate (28.34 mg/g feed).

The discrepant degradation profile observed between XynC and ECXAjEr/ECXAjBr may be attributed to the presence of accessory enzymes in the *A. japonicus* enzymatic cocktails. Teixeira et al. (2023) observed that a complete enzymatic cocktail from *A. niger* ATCC 9642 and *Trichoderma reesei* NRRL 3652 was more effective than commercial enzymes (cellulases and pectinases) in degrading biomasses used in animal nutrition. Similarly, *Aspergillus niveus* 43 produced an enzyme cocktail rich in phytase, protease, and xylanase that was capable of degrading non-ruminant animal diets, showing even better performance than commercial enzymes (de Oliveira Simas et al. 2024).

The synergy between hemicellulase and cellulase is a key strategy for achieving the economically viable bioconversion of lignocellulosic biomass. This is due to the structure of hemicellulose, which is intrinsically associated with cellulose and physically restricts the access of cellulases to cellulose fibers. Therefore, complementary enzymatic activities such as arabinofuranosidases, mannanases, galactosidases, and pectinases are required, and these enzymes are increasingly present in the feed additive market, highlighting the relevance of their study (Sureshkumar et al. 2023). Consequently, the synergistic action through enzymatic cocktails enhances enzyme accessibility to substrates (Brar et al. 2020), which is corroborated in the present study by the increased release of reducing sugars.

Given that the crude extract contained multiple extracellular enzymes, its interaction with the commercial enzyme was evaluated to assess potential synergistic effects. Synergism between enzymes can only be confirmed when the release of target products induced by the enzyme cocktail exceeds the amount released by the individually applied enzymes in the same proportion (Hu et al. 2011). The enzyme cocktail containing multiple extracellular enzymes showed that the partial replacement of the commercial enzyme with ECAjBr (25 U of xylanase, 12.5%) resulted in improvements in diet hydrolysis without increasing the enzyme load. Therefore, it can be inferred that there was synergism between enzymes. This synergistic effect is attributed to the action of enzymes on xylan hydrolysis, which results in increased accessibility to cellulose and other plant cell wall components, corroborating the results reported by Hu et al. (2011).

Xylanases from *Humicola brevis* var. *thermoidea* and *Rasamsonia composticola* exhibited synergistic activity with commercial enzyme cocktails in the degradation of lignocellulosic biomass when used to replace 50% of the commercial preparation (Almeida et al. 2022; Franco et al. 2024). Moreover, a bifunctional xylanase/feruloyl esterase enzyme (XynII-Fae) was used to improve the efficiency of a commercial cellulase preparation in the degradation of different biomasses, resulting in a reducing sugars release of 9.47 ± 0.26 mg/mL from corn straw, 5.84 ± 0.29 mg/mL from wheat straw, and 2.93 ± 0.08 mg/mL from sugarcane bagasse. These results were obtained with the most effective mixture (4% XynII-Fae and 6% commercial cellulase) (Wang et al. 2025).

Overall, these studies demonstrate the potential of using exogenous enzymes as bioactives in animal nutrition. Their mode of action can promote the reduction of feed viscosity through the hydrolysis of polysaccharides fermented by microbial pathogens. Furthermore, nutrient access to the gut microbiota can be improved through the production of prebiotic oligosaccharides, thereby increasing nutrient bioavailability in non-ruminant animal diets (Plouhinec et al. 2023). The application of the developed cocktail in complete poultry diets suggests a potential increase in nutrient bioavailability for these animals.

## Conclusion

This study presents new findings regarding the simultaneous production of phytase and xylanase by *A. japonicus* in a stirred-tank bioreactor, using alternative carbon sources/low-cost agro-industrial residues (wheat bran + soybean bran) to reduce production costs. *A. japonicus* produced high levels of phytase and xylanase in a well-controlled bioreactor environment, resulting in a functionally superior enzyme product and demonstrating high potential for industrial-scale production. Despite the study focused on the production of phytase and xylanase, other enzymes production in a bioreactor was also evaluated, observing an increase in protein production compared to cultivation in Erlenmeyer flasks, enzymes for lignocellulosic biomass degradation, thereby reinforcing the bioreactor’s suitability for generating multifunctional enzyme cocktails. The *A. japonicus* enzymatic cocktail exhibited superior hydrolytic activity on poultry diets compared with commercial enzymes and showed synergistic effects when combined with them. Its application in animal feed outperformed the effects provided by the commercial enzymes alone, even at low concentrations. Overall, the addition of the *A. japonicus* enzymatic cocktail, rich in both core and accessory enzymes, may enhance the beneficial effects of commercial enzymes, leading to increased nutrient bioavailability and potentially improving the performance of non-ruminant animals. 

## Data Availability

No datasets were generated or analysed during the current study.
